# Diagnostic challenges of yellow nail syndrome in an older patient with recurrent pleural effusion: A case report

**DOI:** 10.1097/MD.0000000000045864

**Published:** 2025-11-14

**Authors:** Dalin Di, Qingxiang Zhang, Yanyan Zhang, Chun'e Gao, Zongliang Li, Zhiwen Xu, Yuxia Li

**Affiliations:** a Department of Immunology, School of Basic Medical Sciences, Shandong Second Medical University, Weifang, Shandong, China; b Department of Respiratory Critical Care Medicine, Weifang No. 2 People’s Hospital, Weifang, Shandong, China; c Department of Rheumatology and Immunology, Weifang No. 2 People’s Hospital, Weifang, Shandong, China; d Ultrasound Medicine Department, Weifang No. 2 People’s Hospital, Weifang, Shandong, China; e Key Laboratory of Immune Microenvironment and Inflammatory Disease Research in Universities of Shandong Province, School of Basic Medical Sciences, Shandong Second Medical University, Weifang, Shandong, China.

**Keywords:** older, recurrent pleural effusion, vitamin E, yellow nail syndrome

## Abstract

**Rationale:**

Yellow nail syndrome (YNS) is a rare disorder characterized by a triad of yellow nails, lymphedema, and respiratory manifestations, which primarily presents as recurrent pleural effusion in older patients. Given that all 3 features of the triad may not be present synchronously, diagnosis of YNS poses a significant clinical challenge, especially in elderly populations.

**Patient concerns:**

A 75-year-old male with a history of chronic obstructive pulmonary disease and eczema presented with worsening cough, dyspnea, and recurrent left-sided pleural effusion. Physical examination revealed characteristic yellow nails exhibiting thickening, discoloration, increased curvature, and absence of lunulae and cuticles, along with mild facial and ankle edema.

**Diagnoses:**

YNS was diagnosed based on the presence of characteristic yellow nails, lymphocyte-predominant exudative pleural effusion, and the exclusion of infectious, malignant, and tuberculous etiologies through comprehensive evaluations including pleural fluid analysis, cytology, microbiology, chest imaging, and bronchoscopy.

**Interventions:**

The patient underwent therapeutic thoracentesis with drainage of 4760 mL of pleural fluid over a 20-day period. Anti-infective therapy was administered (initially cefoxitin, later escalated to piperacillin-tazobactam and levofloxacin) along with oral vitamin E supplementation (1200 IU/d) initiated on the fourth day of hospitalization.

**Outcomes:**

Following treatment, the patient experienced gradual improvement in dyspnea with substantial reduction in pleural effusion from 8.25 cm to 0.36 cm in depth. During the long-term follow-up (June 2021–May 2025, approximately 47 months), sustained improvement in respiratory symptoms was observed, with progressive improvement in fingernail morphology, though yellow discoloration partially persisted.

**Lessons:**

This case highlights the importance of considering YNS in the differential diagnosis of recurrent pleural effusion in older patients, even in the absence of typical lymphedema. The improvements observed following vitamin E therapy suggest its potential therapeutic benefit; however, further studies are warranted to establish definitive treatment protocols for this rare condition.

## 1. Introduction

Yellow nail syndrome (YNS) is a rare and often elusive clinical entity that typically presents in individuals aged >50 years old. Its global prevalence is estimated to be <1 in 10,00,000 individuals, making diagnosis a persistent challenge for clinicians.^[1]^ The classic triad of YNS includes nail dystrophy, characterized by slow growth, yellowish discoloration, and thickening, as well as lymphedema and respiratory involvement. Heller first described nail abnormalities in 1927; however, it was Samman and White’s 1964 case series that established the syndromic association between YNS and lymphatic dysfunction, coining the term “YNS.”^[2,3]^ Despite the seemingly straightforward diagnostic criteria, YNS often presents with various clinical manifestations that complicate its diagnosis. In older patients, the diagnostic process is further complicated by atypical presentations. The scarcity of reported cases in which recurrent pleural effusion is the primary presenting feature further exacerbates this significant diagnostic challenge. The exact pathogenesis of YNS remains incompletely understood; however, the prevailing hypothesis centers on lymphatic dysfunction, with genetic and other contributing factors.^[4,5]^ Research suggests that impairment of the Wnt/PCP signaling pathway may disrupt lymphangiogenesis, potentially contributing to the development of YNS.^[6]^ Currently, there is no definitive cure for YNS and management focuses on supportive care. The therapeutic efficacy of interventions, such as vitamin E and octreotide, remains uncertain, underscoring the need for further research. Considering the diagnostic challenges and rarity of YNS, detailed case reports with long-term follow-up remain scarce in the literature, particularly in elderly patients presenting with recurrent pleural effusion as the predominant feature. Here, we present a 75-year-old male with YNS who exhibited recurrent unilateral pleural effusion, requiring systematic exclusion of malignant and infectious etiologies. This case provides: detailed diagnostic strategies for excluding malignancy in elderly patients with atypical YNS presentations, and 47-month follow-up data on sustained clinical improvement with vitamin E therapy, contributing valuable long-term outcome evidence for YNS management in geriatric populations.

## 2. Case presentation

A 75-year-old retired male teacher presented to our hospital in June 2021 with a 10-day history of worsening of a preexisting chronic cough, dyspnea, and sputum production. The patient had a history of recurrent left-sided pleural effusion dating back to 2014, during which time he underwent thoracentesis and experienced symptomatic relief. His 40-year teaching experience involved chronic exposure to classroom dust.

### 2.1. Physical examination and ancillary investigations

**Figure 1.**
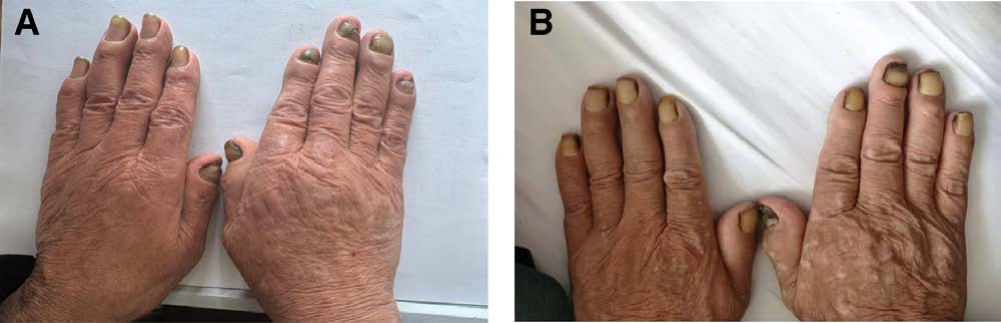
Clinical response of YNS to vitamin E therapy. (A) At presentation, showing classic yellow nail dystrophy, a diagnostic hallmark of YNS. (B) Long-term follow-up post-vitamin E supplementation (May 2025), showing significant improvement in right hand fingernails, despite partial persistence of yellow discoloration. YNS = yellow nail syndrome.

**Figure 2.**
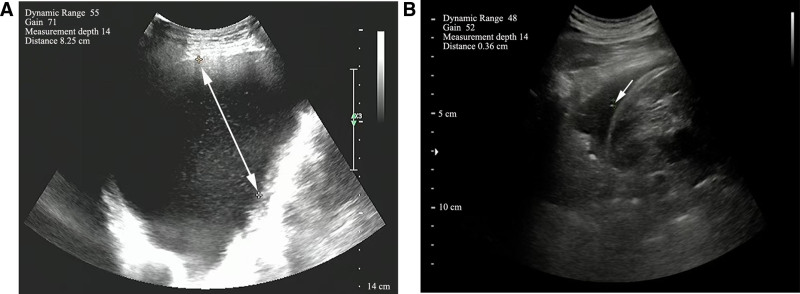
Chest ultrasound images showing pleural effusion before and after treatment. (A) Pretreatment chest ultrasound image showing a large left pleural effusion, measuring 8.25 cm in depth. (B) Posttreatment chest ultrasound image showing near-complete resolution of the left pleural effusion, with a minimal residual depth of 0.36 cm.

**Figure 3.**
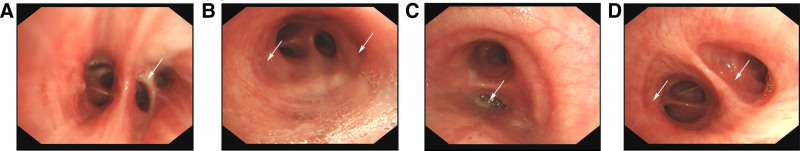
Bronchoscopic findings revealed bronchitis in the left lower lobe bronchus. (A) Right lower lobe basal segment showing significant purulent secretions. (B) Right middle lobe bronchus showing mucosal hyperemia. (C) Left main bronchus orifice showing substantial purulent secretions. (D) Right lower lobe bronchus showing mucosal hyperemia.

The patient presented with a respiratory rate of 21 breaths per minute and a barrel-shaped chest, indicating mild respiratory distress. Auscultation revealed diminished bilateral breath sounds with scattered dry rales on the right side. Mild facial and ankle edema were also noted. Examination of the nails revealed classic yellow nails exhibiting thickening, yellow discoloration, increased curvature, and absence of lunulae and cuticles (Fig. 1). Initial chest ultrasound revealed left pleural effusion, and subsequent imaging confirmed its persistence, measuring 8.25 cm in depth (Fig. 2). Pleural fluid analysis revealed a pale-yellow exudative effusion characterized by lymphocyte predominance, elevated eosinophil levels, elevated glucose (6.58 mmol/L), and decreased total cholesterol (1.94 mmol/L). Microbiological and cytological examinations of the pleural fluid were negative for pathogens and malignant cells, respectively (Table 1). Blood tests indicated mild anemia (hemoglobin: 112 g/L), an increased percentage of eosinophils (7.2%), hypoalbuminaemia (albumin: 34.3 g/L), elevated C-reactive protein (8.82 mg/L), elevated lactate dehydrogenase (167.7 U/L), and reduced levels of total protein (51.1 g/L) and globulin (16.8 g/L; Table 2). Notably, blood gas analysis results were within normal limits, suggesting the absence of significant respiratory failure (Table 3). Bronchoscopy revealed bronchitis (Fig. 3), with bronchoalveolar lavage fluid cytology showing neutrophil predominance and negative microbiological results. Cardiovascular assessments, including echocardiography and electrocardiography, showed largely preserved cardiac function with only mild valvular regurgitation. Pro-B-type natriuretic peptide and D-dimer levels were within the normal range (0–300 pg/mL and 0–0.5 ug/mL respectively), and tumor markers were negative. Collectively, these findings led to the final diagnosis of YNS complicated by left pleural effusion and chronic obstructive pulmonary disease (COPD) with acute lower respiratory tract infection.

**Table 1:** Pleural fluid analysis results.

Test	Pleural fluid	Serum	Ratio	Reference range
Biochemical parameters				
Total protein (g/L)	32.7	51.1	0.64	Exudate > 0.5
LDH (U/L)	161	167.7	0.96	Exudate > 0.6
Glucose (mmol/L)	6.58 ↑	4.66	1.41	3.6–5.5
Total cholesterol (mmol/L)	1.94 ↓	–	–	2.32–5.18
Triglycerides (mmol/L)	0.19	–	–	0–1.24
Amylase (U/L)	29.78	–	–	0–450
ADA (U/L)	8.9	–	–	0–25
Cell count and differential				
Total cell count (×10⁴/mL)	120	–	–	–
Lymphocytes (%)	78.4 ↑	–	–	<50
Neutrophils (%)	0	–	–	Variable
Eosinophils (%)	16.8 ↑	–	–	<10
Macrophages (%)	4.8	–	–	–
Other characteristics				
Appearance	Yellow, slightly turbid	–	–	–
Cytology	Negative for malignancy	–	–	–
Bacterial culture	Negative	–	–	–
Xpert MTB/RIF, AFB	Negative	–	–	–

ADA = adenosine deaminase, AFB = acid-fast bacilli, g/L = grams per liter, LDH = lactate dehydrogenase, mL = milliliter, mmol/L = millimoles per liter, TB = tuberculosis, U/L = units per liter, Xpert MTB/RIF = molecular test for mycobacterium tuberculosis and rifampicin resistance.

**Table 2:** Blood laboratory results.

Test	Result	Reference range
WBC	5.58	3.5–10.0 × 10^9^
% Eosinophils	7.2 ↑	0.5–5.0%
Haemoglobin	112 ↓	130–175 g/L
Total protein	51.1 ↓	65–85 g/L
Globulin	16.8 ↓	20–40 g/L
Platelet	240	100–300 × 10^9^
LDH	167.7 ↑	120–150 U/L
Albumin	34.3 ↓	40–55 g/L
CRP	8.82 ↑	0–2.1 mg/L
Glucose	4.66	3.9–6.1 mmol/L

CRP = C-reactive protein, g/L = grams per liter, LDH = lactate dehydrogenase, mg/L = milligrams per liter, U/L = units per liter, WBC = white blood cell.

**Table 3:** Blood gas analysis results.

Test	Result	Reference range
pCO_2_	41 mm Hg	35–45 mm Hg
pO_2_	91 mm Hg	80–100 mm Hg
pH	7.39	7.35–7.45

mm Hg = millimeters of mercury, pCO_2_ = partial pressure of carbon dioxide, pO_2_ = partial pressure of oxygen.

### 2.2. Patient treatment

Initially, the patient underwent therapeutic thoracentesis and drainage, yielding 4760 mL of pleural fluid over a 20-day period. Given the patient’s presentation with acute exacerbation of COPD and a lower respiratory tract infection, empirical antibiotic therapy with cefoxitin (2.0 g intravenously every 12 hours) was initiated to target potential bacterial pathogens. Subsequently, due to the persistent pleural effusion and lack of clinical resolution of infectious symptoms, the antibiotic regimen was escalated to piperacillin-tazobactam (4.5 g intravenously every 12 hours) and levofloxacin (0.5 g intravenously once daily). In addition to antibiotic therapy, oral vitamin E supplementation (1200 IU/d) was commenced on the fourth day of hospitalization to treat the YNS. During hospitalization, a comprehensive cardiovascular assessment including echocardiography, electrocardiography, and measurement of pro-B-type natriuretic peptide and D-dimer levels was performed. These evaluations indicated that mild valvular regurgitation was not clinically significant in the context of the current presentation and did not warrant specific cardiovascular interventions. Following these treatments, the patient exhibited gradual improvement in dyspnea, accompanied by a reduction in pleural effusion volume. Levofloxacin was discontinued after 7 days, contingent upon sustained clinical improvement and a 72-hour afebrile status. Piperacillin-tazobactam was continued until July 13, 2021, when the patient was discharged. Prior to discharge, a comprehensive reassessment of pleural effusion was conducted using chest ultrasonography, and revealed a minimal residual pleural effusion, measuring 0.36 cm in depth (Fig. 2), suggestive of trace effusion with limited clinical relevance. Integrating the findings from both imaging modalities confirmed a substantial reduction in pleural effusion and marked overall clinical improvement. However, the yellow nail showed no significant change from baseline at the time of discharge, and the patient continued oral vitamin E as maintenance therapy.

### 2.3. Outcome and follow-up

The patient tolerated all treatments well during hospitalization without adverse events. During the extended follow-up period from July 2021 through May 2025 (47 months), the patient maintained oral vitamin E therapy (1200 IU/d) with excellent tolerability. Nail dystrophy showed progressive improvement, particularly in the right hand. No recurrence of significant pleural effusion was observed, and respiratory symptoms remained stable. The patient continues on vitamin E therapy with an overall stable condition.

### 2.4. Ethics statement

All consent procedures and details were approved by our institution’s institutional review board (approval number: KY2025-091-01). Written informed consent was obtained from the patient for publication.

## 3. Discussion

YNS is a rare and diagnostically elusive disorder that primarily relies on clinical assessment for diagnosis. Characterized by the classic triad of yellow nail dystrophy, lymphedema, and respiratory involvement, YNS presents with considerable diagnostic challenges. This complexity arises from the infrequent synchronous presentation of all 3 cardinal features; consequently, the presence of only 2 of the 3 criteria is deemed sufficient to establish a diagnosis.^[7]^ The present case report provides meticulous details of YNS in an older patient, distinguished by recurrent left-sided pleural effusion as the salient initial and principal clinical manifestation, which was further complicated by preexisting COPD and eczema. This unique constellation of findings warrants a focused discussion on several key aspects: the inherent diagnostic difficulties associated with YNS, the clinical significance of recurrent pleural effusion as a presenting feature within this uncommon syndrome, and the therapeutic dilemmas posed by the absence of a definitive curative treatment.

This patient clearly fulfilled the diagnostic criteria for YNS. First, the patient exhibited hallmark nail dystrophy, with nails demonstrating diffuse yellow discoloration, thickening, and accentuated curvature of the nail plate. Pathogenic nail changes are widely recognized as the most diagnostically significant feature of YNS.^[8]^ Second, recurrent left-sided pleural effusion was a prominent feature that served as the primary clinical manifestation during hospitalization. Pleural fluid analysis was consistent with YNS, revealing a pale-yellow exudative effusion with lymphocyte predominance and an elevated eosinophil count. While YNS-associated pleural effusions are typically exudative and frequently bilateral with lymphocyte predominance.^[1]^ The unilateral and recurrent nature in this patient may represent an atypical variant. Third, although overt lymphedema was absent at the initial presentation, the development of mild facial and ankle edema during hospitalization suggested the presence of YNS-related lymphatic involvement. Although subtle and intermittent, this finding provides crucial corroborative evidence. Finally, the patient’s preexisting COPD and findings of bronchitis aligned with the respiratory manifestations commonly observed in YNS. Concurrent acute lower respiratory tract infections, potentially representing COPD exacerbations, may also be considered within the spectrum of respiratory manifestations associated with YNS.

The diagnosis of YNS, particularly in older patients presenting with pleural effusion as the primary symptom, is fundamentally a process of exclusion, given the absence of pathognomonic diagnostic tests. Therefore, a meticulous differential diagnosis was essential. To definitively diagnose YNS in this case, other common causes of pleural effusion were systematically excluded. First, tuberculous pleurisy was excluded owing to the finding of normal pleural fluid adenosine deaminase levels and negative results from comprehensive tuberculosis-related etiological investigations, including acid-fast bacilli smears, molecular test for mycobacterium tuberculosis and rifampicin resistance, and *Mycobacterium tuberculosis* DNA analysis. Subsequently, the likelihood of malignant pleural effusion significantly diminished. While pleural fluid cytology and bronchoscopic brushing yielded no evidence of malignant cells, and tumor marker levels were unremarkable, malignancy remained a consideration until these investigations proved negative. Parapneumonic effusion was considered unlikely because although the patient exhibited clinical signs of pulmonary infection, such as bronchitis, the persistent and slow-resolving nature of the pleural effusion, even with anti-infective treatment, was inconsistent with the typically rapid resolution observed in uncomplicated parapneumonic effusions. Cardiogenic pleural effusion and pulmonary embolism were considered less probable. Echocardiography and electrocardiography revealed essentially normal cardiac function with only mild valvular regurgitation. Pro-BNP and D-dimer levels were within normal limits and blood gas analysis showed no significant abnormalities, further reducing the likelihood of these conditions. Ultimately, following this rigorous process of exclusion and integration of the patient’s distinctive clinical features, including pathognomonic yellow nail dystrophy and recurrent lymphocyte-predominant exudative pleural effusion, a diagnosis of YNS was confidently established.

Following standard pleural effusion guidelines, we recommend a stepwise diagnostic approach for suspected YNS-associated pleural effusion, where each stage has clear stopping criteria. Comprehensive pleural fluid analysis should be performed first, including specific biomarkers such as adenosine deaminase levels, lactate dehydrogenase, cytological examination, and tumor marker assessment, particularly for conditions such as lung adenocarcinoma and mesothelioma. If this initial analysis establishes a definitive diagnosis, further invasive procedures are unnecessary. For cases requiring further evaluation, serial imaging studies should be performed beginning with chest ultrasonography and CT imaging. Thoracoscopic biopsy should be reserved for cases where noninvasive investigations remain inconclusive. This approach balances diagnostic certainty against procedural risk while avoiding unnecessary invasive procedures.

Lymphatic dysfunction, particularly impaired lymphatic drainage, is currently the predominant pathogenic theory for YNS. Lymphocyte count alterations have been recognized as important biomarkers in various systemic inflammatory and immune-mediated conditions.^[9]^ This lymphatic impairment is considered a central mechanism in the development of the classic triad of the syndrome: nail dystrophy, lymphedema, and pleural effusion.^[10,11]^ However, it is important to acknowledge that etiological research on YNS remains limited, particularly concerning the direct evidence of genetic, environmental, and lifestyle contributions. Although investigations suggest a potential role for genetic factors in YNS development, with familial aggregation cases indicating a degree of genetic predisposition, the precise underlying genetic mechanisms require further elucidation.^[12,13]^ Environmental factors are also considered potential contributors to YNS. In this particular case, the patient’s 40-year career as a teacher involved chronic exposure to classroom dust, raising the possibility of a correlation between occupational exposure and YNS development, potentially affecting lymphatic function. However, a definitive causal link and the specific pathophysiological pathways involved require further investigation. Currently, YNS lacks a definitive curative treatment, and management strategies are primarily focused on symptomatic and supportive care.^[14]^

The therapeutic approach for this patient incorporated several key strategies, including thoracentesis for prompt symptomatic relief, empirical antibiotic administration to manage the presumed pulmonary infection, and oral vitamin E supplementation. This multimodal strategy aligns with the findings of Maldonado et al, who indicated that respiratory manifestations associated with YNS are often effectively managed through pharmacological and surgical interventions.^[15]^ Vitamin E has been proposed as a potential therapeutic agent for YNS with previous case reports suggesting its potential efficacy. The proposed mechanisms of action include antioxidant properties, potential to improve lymphatic function, and anti-inflammatory effects. However, robust confirmation of the efficacy of vitamin E from high-quality randomized controlled trials (RCTs) is lacking and clinical responses appear to vary across reported cases.^[16,17]^ Notably, RCTs that specifically investigated vitamin E levels in childhood YNS have yielded negative results.^[18]^ Despite the absence of definitive evidence from RCTs, vitamin E remains a frequently employed supportive treatment in clinical practice for YNS, primarily because of its perceived potential benefits and the current lack of established curative therapies. In this case, although we observed improvements in the patient’s symptoms and pleural effusion following the initiation of vitamin E treatment, definitively isolating the specific contribution of vitamin E from the combined effects of thoracentesis, antibiotic therapy, and natural remission of the condition proved challenging. Nevertheless, our long-term follow-up observations over nearly 4 years suggest potential sustained benefits of vitamin E supplementation, particularly in the progressive improvement of nail morphology and prevention of pleural effusion recurrence.

The mechanistic pathways linking vitamin E supplementation to improved lymphatic drainage and nail matrix function in YNS remain incompletely understood. Several theoretical mechanisms may contribute: vitamin E’s antioxidant properties may reduce oxidative stress-induced damage to lymphatic endothelium, its anti-inflammatory effects may modulate cytokine expression affecting lymphangiogenesis, and it may influence cellular membrane stability affecting lymphatic vessel integrity. Recent research has identified impaired Wnt/PCP signaling in YNS pathogenesis, which may represent a potential target for therapeutic intervention.^[6]^ Future prospective clinical trials should employ multicenter randomized controlled designs comparing high-dose vitamin E against placebo, with primary endpoints including objective measures of nail morphology and respiratory function, though such studies face challenges due to YNS rarity and ethical considerations.

Previous case reports suggested that octreotide may be effective in managing refractory pleural effusion or lymphedema associated with YNS. The proposed mechanisms involve the reduction of lymphatic fluid production or enhancement of lymphatic vessel function.^[19–21]^ However, the efficacy and safety profile of octreotide in YNS require further validation through high-quality RCTs. Furthermore, potential adverse effects, including gastrointestinal disturbances, such as diarrhea and nausea, and the relatively high cost of therapy, warrant careful consideration. In this case, given the satisfactory disease control achieved with the conservative management strategy, more aggressive interventions, including octreotide therapy, were not pursued.

This case report emphasizes several important clinical implications. First, it highlights recurrent pleural effusion as a significant respiratory manifestation in older patients with YNS, suggesting that clinicians should consider YNS in the differential diagnosis of unexplained recurrent pleural effusion in older patients. Second, this case reemphasizes the diagnostic challenges of YNS. YNS has diverse clinical manifestations and lacks specific diagnostic indicators, and the diagnosis mainly relies on clinical features and exclusion. Third, this case adds to the limited evidence for the treatment of YNS, especially the application of vitamin E, suggesting that vitamin E may have potential therapeutic value, although its exact efficacy needs to be confirmed by further high-quality research. Finally, this case reminds clinicians that even in the context of common diseases such as COPD, they should remain vigilant about rare diseases such as YNS to achieve early recognition, timely diagnosis, and standardized treatment, and ultimately improve patient prognosis. Given the rarity of YNS and the unique nature of this case, future research should prioritize the following key areas. First, rigorous multicenter RCTs are essential to evaluate the efficacy and safety of pharmacological interventions such as vitamin E in the treatment of YNS. These trials should aim to determine optimal treatment regimens, including dosage, duration, and target patient populations. Second, in-depth investigations into the pathogenesis of YNS are warranted. This includes studies that aim to elucidate the molecular mechanisms underlying lymphatic dysfunction, explore genetic predispositions, assess the influence of environmental factors, and identify novel therapeutic targets. Moreover, studies that involve comprehensive assessments of lymphatic dysfunction with the use of imaging modalities such as lymphangiography and near-infrared fluorescence lymphatic imaging, genetic analyses, quality of life research, and the establishment of a YNS-specific rare disease registry are critical for expanding the evidence base and advancing diagnostic and therapeutic strategies. Given the rarity of YNS, epidemiological studies are urgently required to ascertain its incidence, prevalence, and potential risk factors. These studies will provide a robust scientific foundation for effective disease prevention and management.

## 4. Conclusion

This case report highlights an exceptionally rare presentation of YNS in an older male, establishing recurrent pleural effusion as a critical and often-overlooked feature. Preexisting COPD further complicates the diagnosis of YNS, underscoring the need for clinicians to consider YNS in older patients with unexplained recurrent pleural effusions, especially in those with yellow nail dystrophy. In terms of treatment, vitamin E shows therapeutic promise but requires rigorous validation. Presenting this unique, challenging case aims to improve clinical recognition of YNS, particularly atypical respiratory presentations in the older population, drive future research into its pathogenesis, and optimize evidence-based treatments for this enigmatic syndrome.

## Author contributions

**Conceptualization:** Dalin Di, Qingxiang Zhang, Zongliang Li, Yuxia Li.

**Data curation:** Dalin Di, Chun’e Gao.

**Investigation:** Chun’e Gao.

**Project administration:** Yuxia Li.

**Resources:** Zongliang Li.

**Supervision:** Qingxiang Zhang, Zhiwen Xu, Yuxia Li.

**Visualization:** Qingxiang Zhang.

**Writing – original draft:** Dalin Di, Yanyan Zhang.

**Writing – review & editing:** Zhiwen Xu, Yuxia Li.
